# Supplementary material for: Mild Therapeutic Hypothermia Protects from Acute and Chronic Renal Ischemia-Reperfusion Injury in Mice by Mitigated Mitochondrial Dysfunction and Modulation of Local and Systemic Inflammation
Source: Int J Mol Sci. 2022 Aug 17;23(16):9229. doi: 10.3390/ijms23169229 (PMC9409407; doi:10.3390/ijms23169229)
Supplement: Supplementary file 1 [file ijms-23-09229-s001.zip › ijms-1854131-supplementary.pdf]

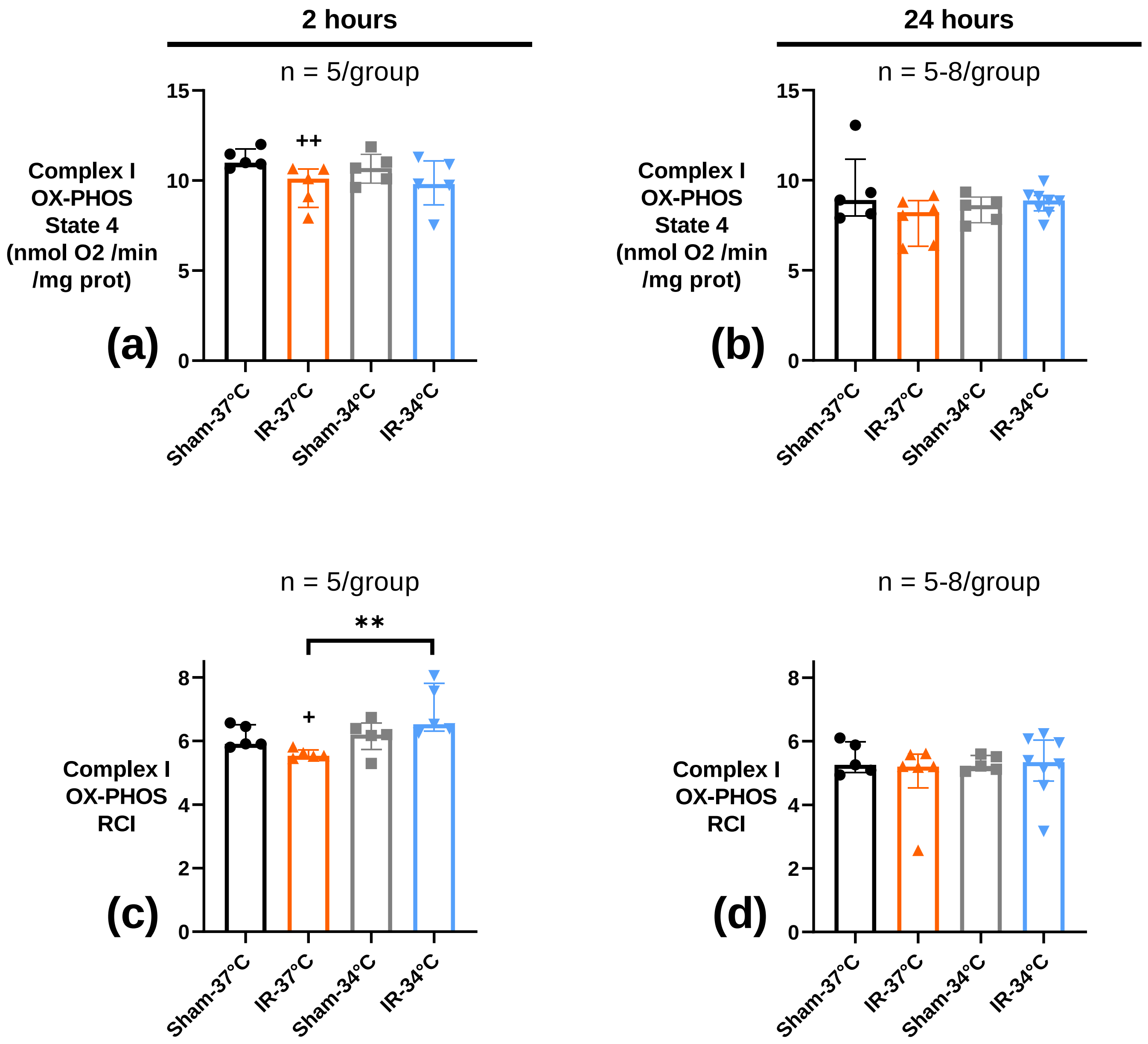

Supplemental Figure S1: Oxidative Phosphorylation (OX-PHOS) in kidney mitochondria after 20 minutes of bilateral renal ischemia where the mice body temperature was maintained at normothermia (IR-37°C) or at mild therapeutic hypothermia (mTH) (IR-34°C), and 2 hours or 24 hours of reperfusion, or after a sham procedure with the same body temperature (Sham-37°C and Sham-34°C). Activity rates of electron transport chain (ETC) complex I were measured through O2 consumption of 250 µg kidney mitochondria: state 4 represents the maximal oxygen consumption after adenosine diphosphate (ADP) has been entirely transformed into adenosine triphosphate (ATP) in excess presence of the substrates of complex I, i.e. after state 3 is over. Respiratory coupling index (RCI) is the calculated (state 3/state 4) ratio, reflecting the tightness of the coupling between respiration and phosphorylation, that is the quantity of O_2 consumed in order to produce ATP. Data are shown as median with interquartiles. ** $p < 0.01$, Mann-Whitney test. + $p < 0.05$, ++ $p < 0.01$ vs Sham-37°C, Mann-Whitney test.

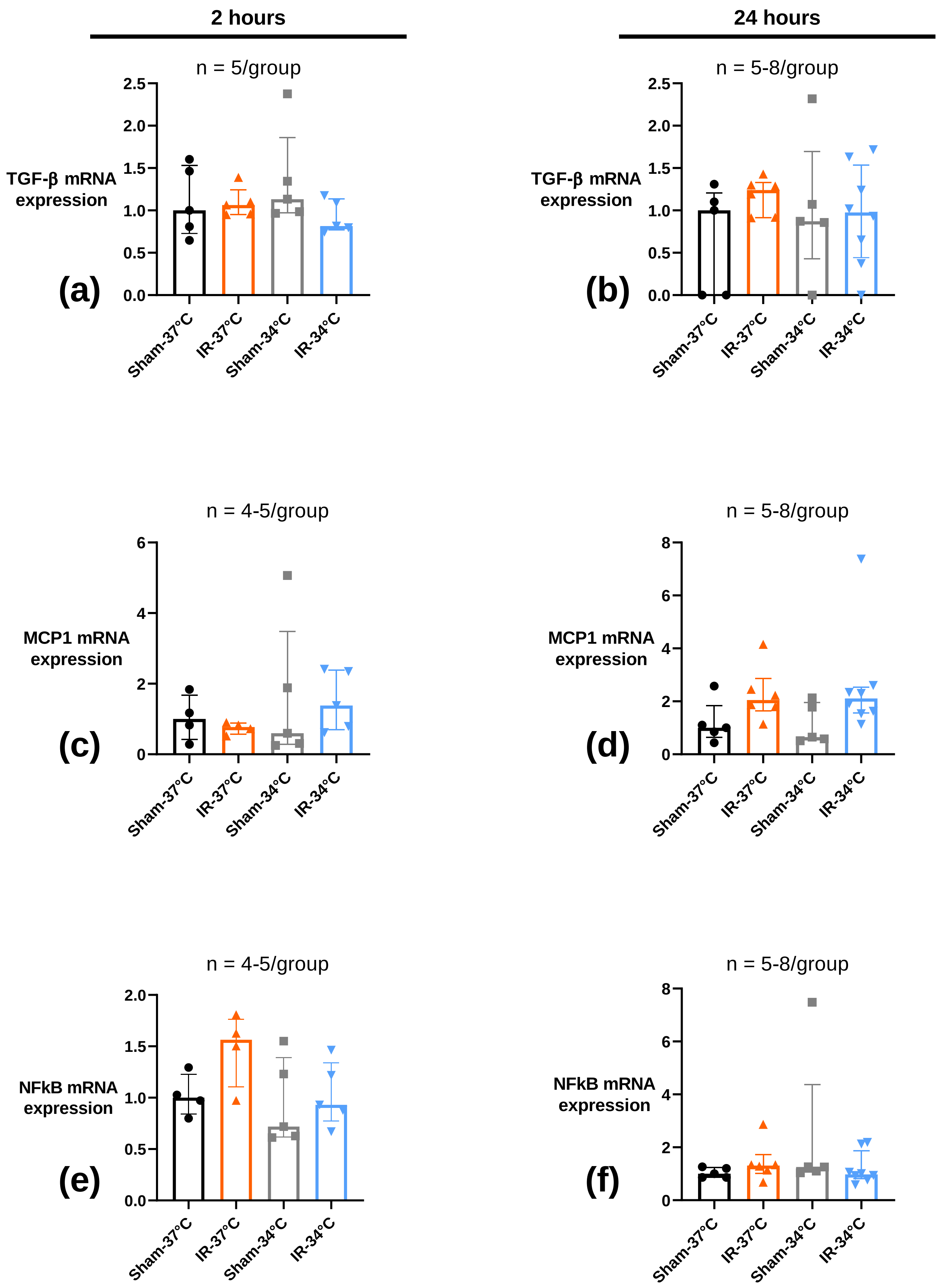

2 hours
24 hours
n = 5/group
TGF-β mRNA expression
(a)
n = 5-8/group
(b)
n = 4-5/group
MCP1 mRNA expression
(c)
n = 5-8/group
(d)
n = 4-5/group
NFkB mRNA expression
(e)
n = 5-8/group
(f)
Sham-37°C
IR-37°C
Sham-34°C
IR-34°C

Supplemental Figure S2: mRNA expression of inflammatory markers TGF-β, MCP1 and NFkB in kidney after 20 minutes of bilateral renal ischemia where the mice body temperature was maintained at normothermia (IR-37°C) or at mild therapeutic hypothermia (mTH) (IR-34°C), and 2 hours or 24 hours of reperfusion, or after a sham procedure with the same body temperature (Sham-37°C and Sham-34°C). Actin mRNA was used as an internal standard to normalize the abundance of cDNA in each sample to normalize results.
Data are shown as median with interquartiles.

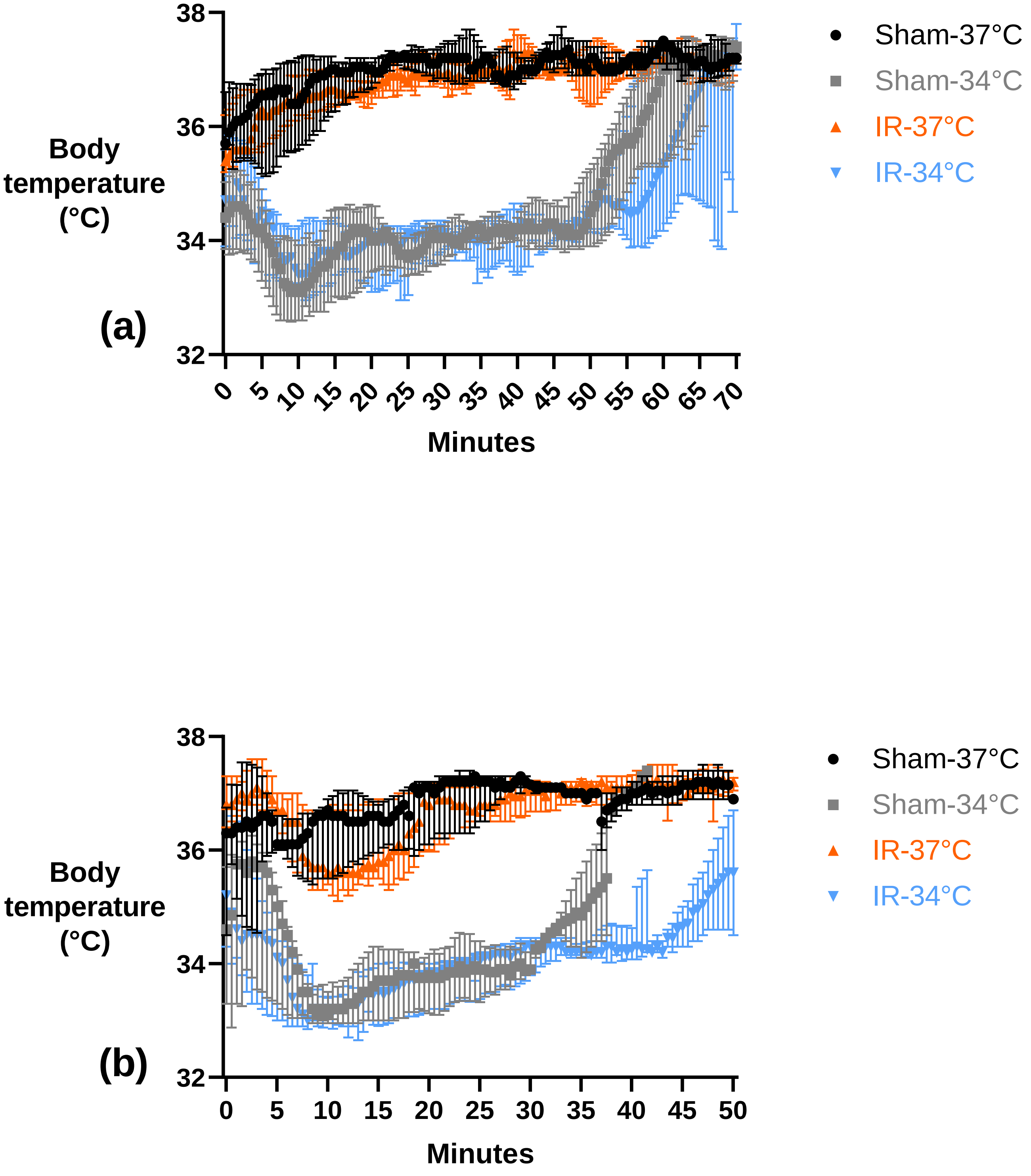

Supplemental Figure S3: Core body temperature measured in mice with an intrarectal probe during the general anesthesia during protocol 1 (a) and protocol 2 (b). After general anesthesia, mice were installed on a homeothermic pallet unit to maintain their temperature either at normothermia (37°C), or mild therapeutic hypothermia (34°C). After stabilization of the temperature, the surgery was performed: protocol 1: 20 minutes of bilateral renal ischemia **(a)**, or protocol 2: 15 minutes of unilateral renal ischemia **(b)**, or a sham procedure at the chosen temperature. After the surgery and as the mice progressively woke up, temperature rose back to normothermia.

Temperature was continuously monitored but measures were recorded every 30 seconds.
Data are shown as median with interquartiles.

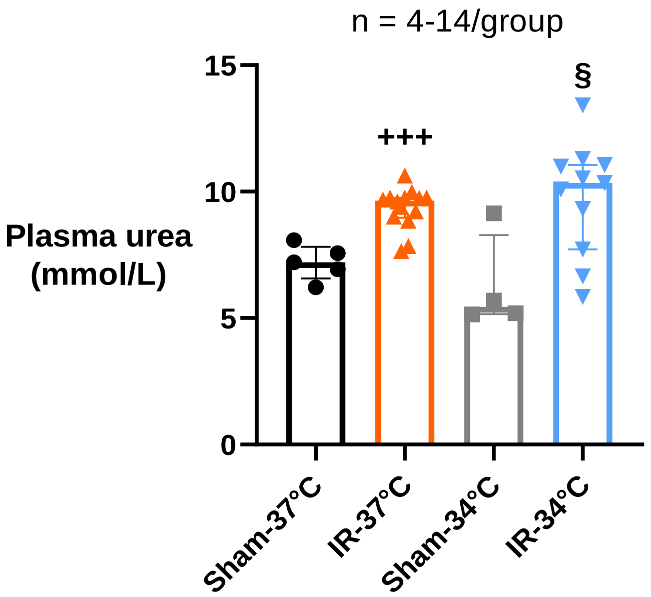

Supplemental Figure S4: Renal function assessed by plasma urea titration after 15 minutes of unilateral renal ischemia where the mice body temperature was maintained at normothermia (IR-37°C) or at mild therapeutic hypothermia (mTH) (IR-34°C), followed by 1 month of reperfusion, or after a sham procedure with the same body temperature (Sham-37°C and Sham-34°C). Data are shown as median with interquartiles. +++ $p < 0.001$ vs Sham-37°C, § $p < 0.05$ vs Sham-34°C, Mann-Whitney test.

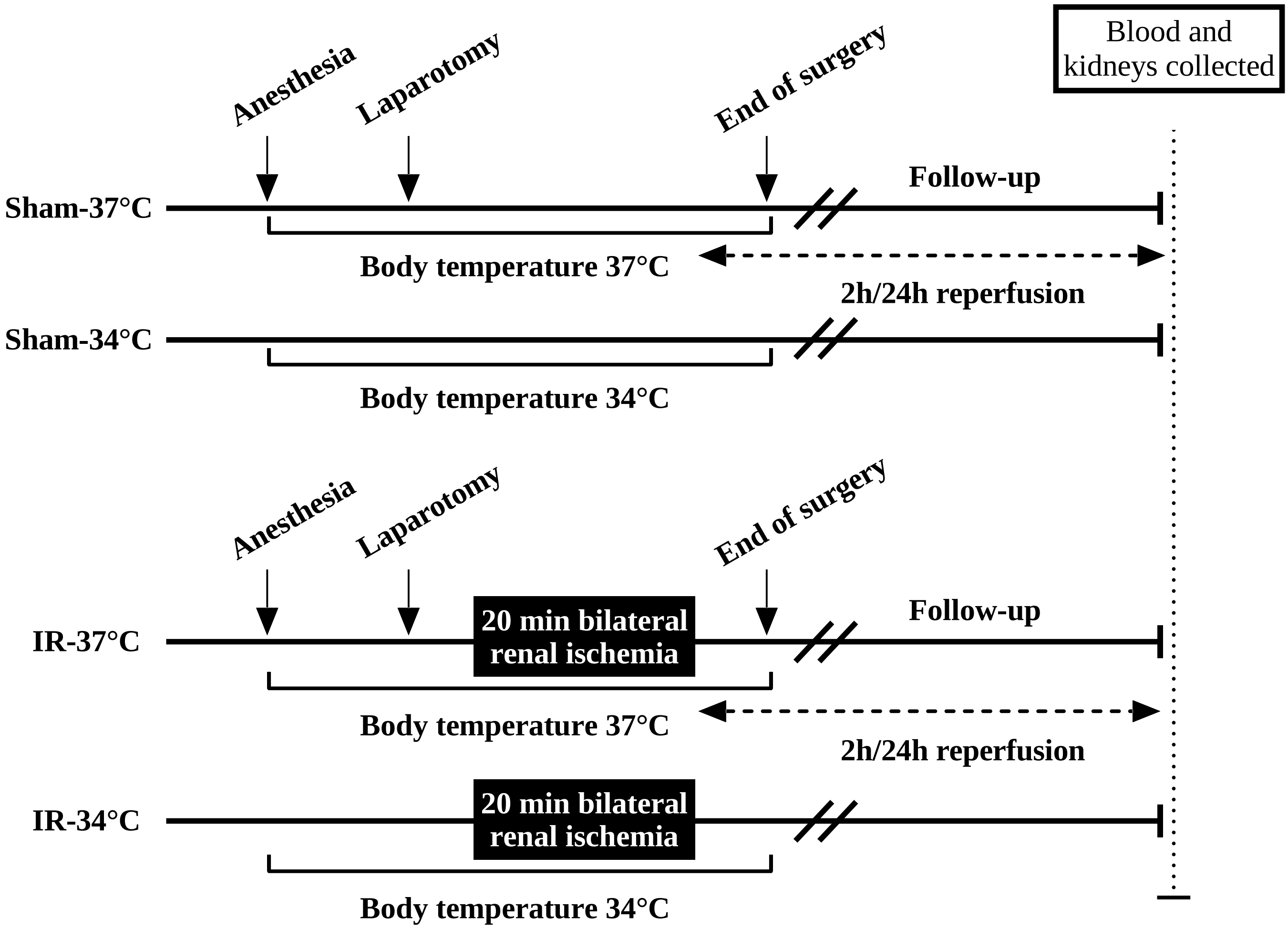

Supplemental Figure S5: Experimental design of protocol 1.

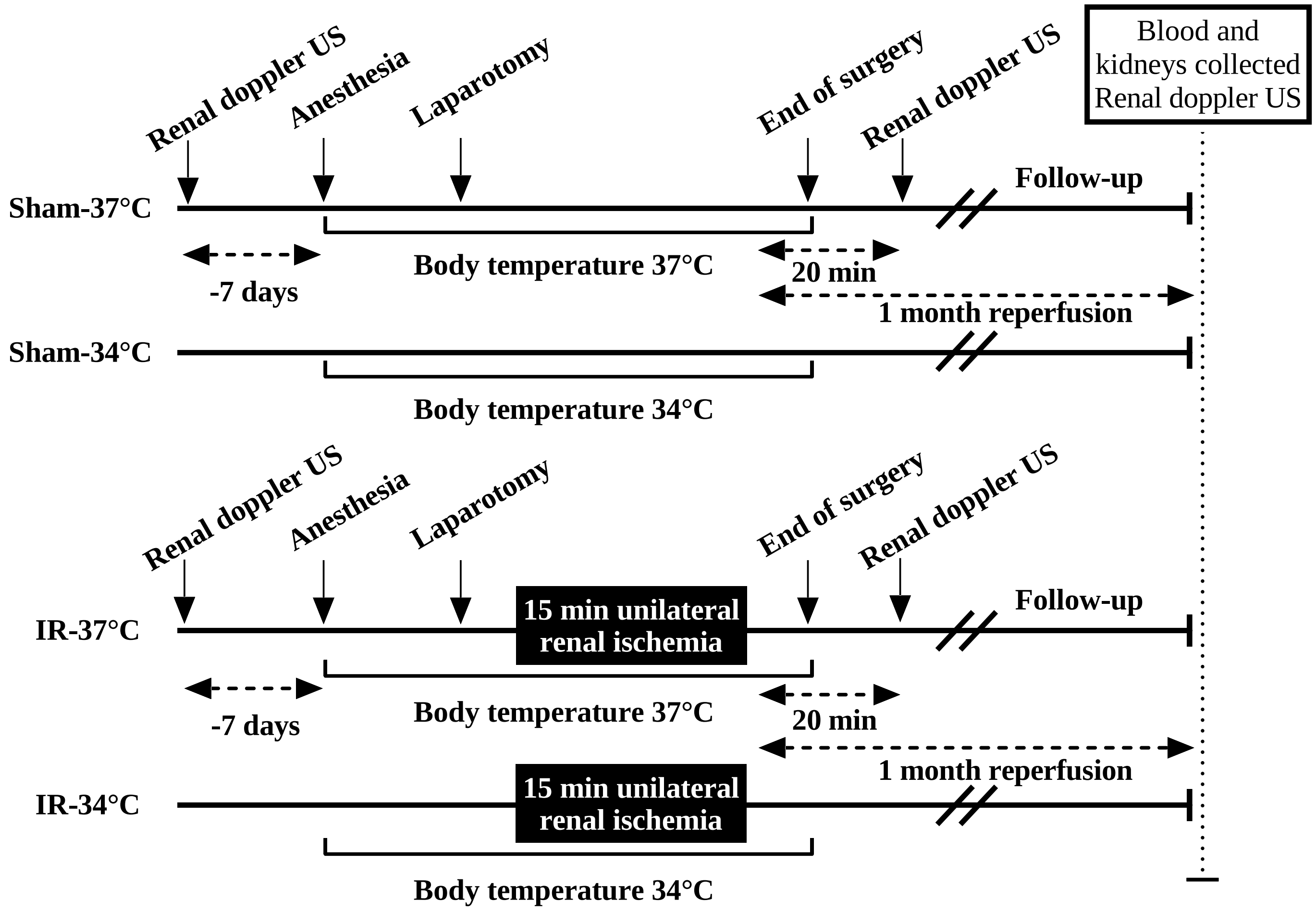

Supplemental Figure S6: Experimental design of protocol 2.

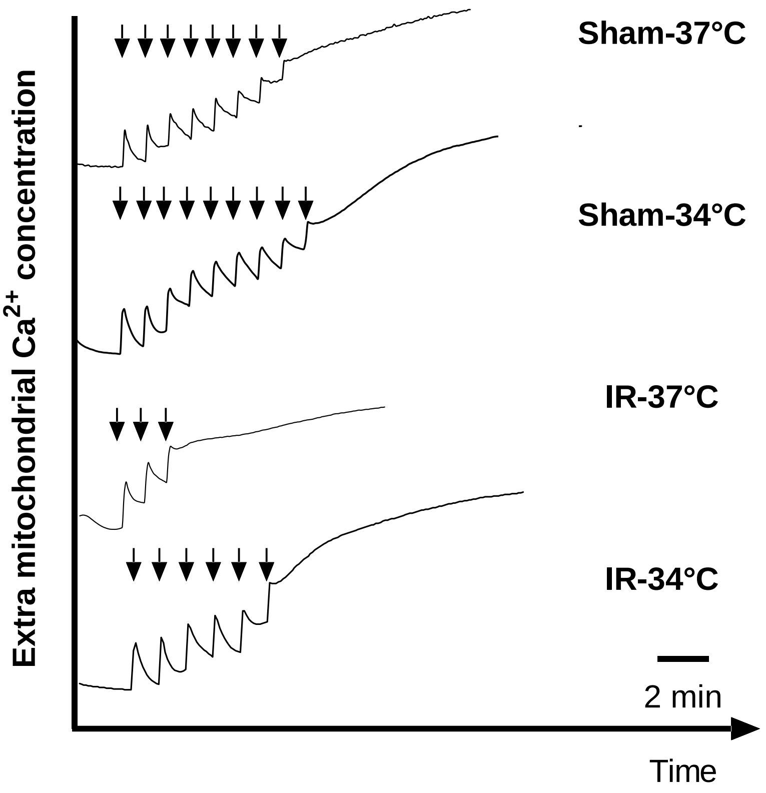

Supplemental Figure S7: Calcium retention capacity (CRC). Representative recordings of Ca^{2+}-induced mitochondrial permeability transition pore (mPTP) opening in each group. Ten nmol of $CaCl_2$ were injected every minute in the isolated mitochondria suspension (represented by a vertical arrow), corresponding to a peak of extramitochondrial Ca^{2+} concentration. Ca^{2+} was quickly taken up by the mitochondria, leading to a return of Ca^{2+} concentration nearly to baseline level. When mPTP opened, the mitochondria released massive Ca^{2+} quantity, corresponding to the abrupt increase of extramitochondrial Ca^{2+} concentration. The CRC is the amount of Ca^{2+} necessary to open the mPTP and an indicator of the resistance of the mitochondria to calcium load. For example, in the Sham-37°C group, 8 pulses of 10 nmol of CaCl2 (80 nmol in total) were necessary to open the mPTP for 250 µg of protein, that is 320 nmol of Ca2+ for 1 mg of protein.

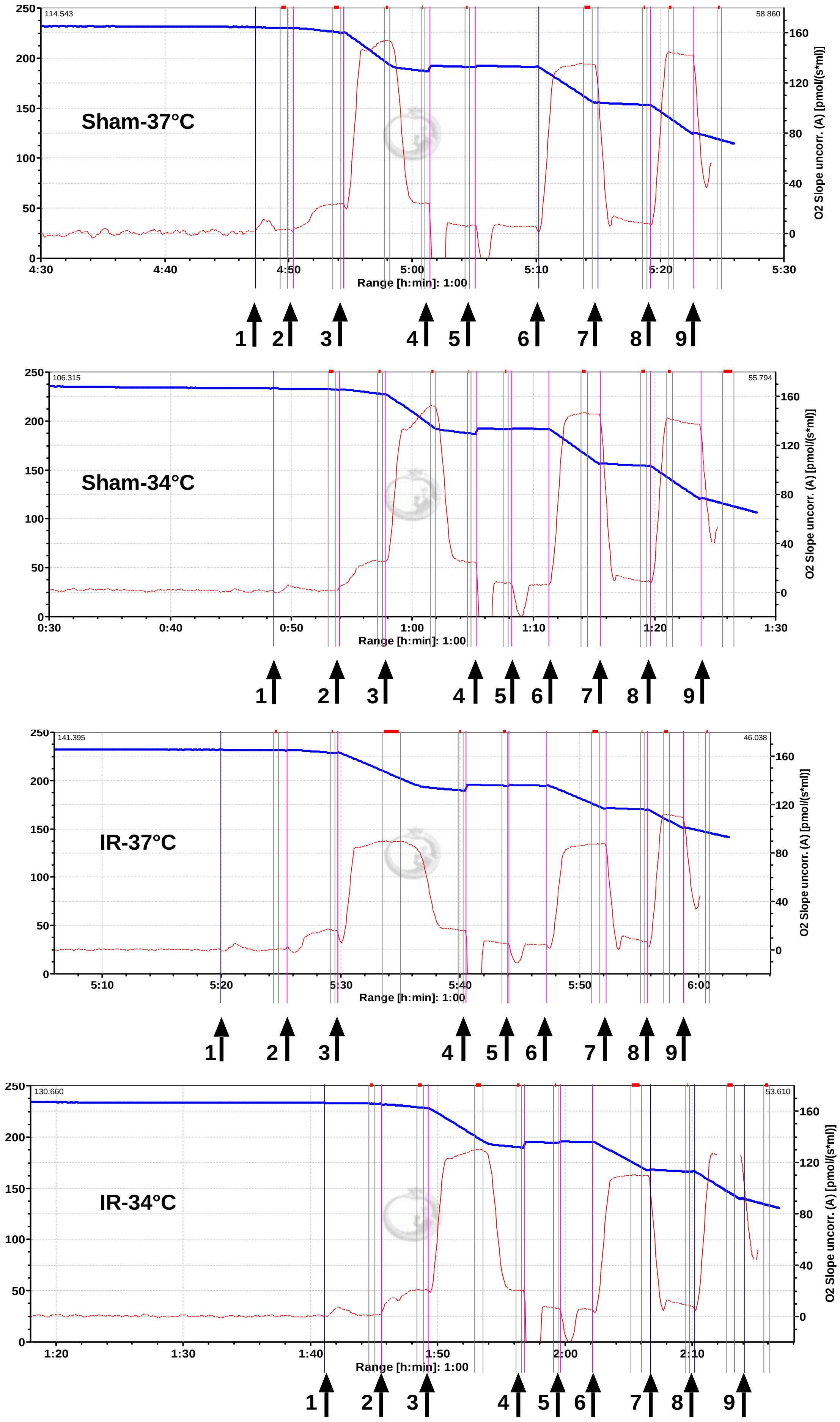

Sham-37°C
114.543
58.860
4:30
4:40
4:50
5:00
5:10
5:20
5:30
Range [h:min]: 1:00
O2 Slope uncorr. (A) [pmol/(s*ml)]
1 2 3 4 5 6 7 8 9
Sham-34°C
106.315
55.794
0:30
0:40
0:50
1:00
1:10
1:20
1:30
Range [h:min]: 1:00
O2 Slope uncorr. (A) [pmol/(s*ml)]
1 2 3 4 5 6 7 8 9
IR-37°C
141.395
46.038
5:10
5:20
5:30
5:40
5:50
6:00
Range [h:min]: 1:00
O2 Slope uncorr. (A) [pmol/(s*ml)]
1 2 3 4 5 6 7 8 9
IR-34°C
130.660
53.610
1:20
1:30
1:40
1:50
2:00
2:10
Range [h:min]: 1:00
O2 Slope uncorr. (A) [pmol/(s*ml)]
1 2 3 4 5 6 7 8 9

Supplemental Figure S8: Oxidative Phosphorylation (OX-PHOS) or mitochondrial respiration. Representative recordings of extramitochondrial O_2 concentration (blue slope) and its derivative (red slope) corresponding to the rate of O2 consumption, *i.e.* mitochondrial respiration, in each group. Mitochondria (250 µg proteins) were incubated (Arrow 1), with 2 mM adenosine diphosphate (ADP) (Arrow 2). Complex I substrates Pyruvate/Malate/Glutamate (5/5/20 mM) were then injected (Arrow 3), the following strong oxygen consumption represents state 3 of complex I. When ADP was entirely phosphorylated (with a decrease in oxygen consumption), state 4 of complex I was reached, allowing to calculate the state 3/state 4 ratio named Respiratory Coupling Index (RCI). An addition of 0.5 µM of Rotenone was then injected (Arrow 4) to inhibit the complex I activity, and ADP in excess and complex II substrate Succinate (10 mM) were added (Arrow 5 and 6) to stimulate state 3 of complex II. Then an addition of 40 µM TTFA was used (Arrow 7) to inhibit the complex II activity, and complex IV substrates TMPD/Ascorbate (0.3/3 mM) were added (Arrow 8) to stimulate state 3 of complex IV. Finally, an addition of 15 mM Azide was used to inhibit the complex IV activity (Arrow 9).
